# Predictors of anemia in a multi-ethnic chronic kidney disease population: a case–control study

**DOI:** 10.1186/s40064-015-1001-z

**Published:** 2015-05-20

**Authors:** Bing Chang Vincent Lau, Kheng Yong Ong, Chun Wei Yap, Anantharaman Vathsala, Priscilla How

**Affiliations:** Department of Pharmacy, Faculty of Science, National University of Singapore, Singapore, Singapore; Department of Medicine (Division of Nephrology), National University Hospital, Singapore, Singapore

**Keywords:** Anemia, Risk factors, Chronic kidney disease, Hemoglobin, Iron

## Abstract

Anemia is a common complication of chronic kidney disease (CKD). However, risk factors of anemia in CKD patients in Singapore are not well established. Hence, a retrospective, case–control study involving non-dialysis CKD patients was conducted to determine possible predictors of anemia in the local CKD population.

Non-dialysis adult CKD patients, not receiving renal replacement therapy or erythropoiesis-stimulating-agents were included. Parameters collected included demographics *e.g.* age, sex and race; clinical data *e.g.* CKD stage and medical/medication histories; and laboratory data *e.g.* serum electrolytes, urinary and hematologic parameters. Patients were classified as anemic or non-anemic using a threshold hemoglobin level of 10 g/dL. The parameters were evaluated for their predictive value for anemia development using multivariate logistical regression and calculation of odds ratios. Statistical analyses were performed using STATA.

A total of 457 patients (162 anemic and 295 non-anemic) were analysed. Multivariate analysis showed that probability of developing anemia was greater for patients with stage 5 CKD (OR 16.76, *p* < 0.001), with hematological disorders (OR 18.61, *p* < 0.001) and with respiratory disorders (OR 4.54, *p* = 0.004). The probability of developing anemia was lower for patients with higher previous hemoglobin concentration (OR 0.32, *p* < 0.001) and in those receiving iron supplements (OR 0.44, *p* = 0.031). Gender and race were not found to be significant predictors of anemia.

Risk of anemia is increased in patients with advanced CKD, haematological disorders, respiratory disorders, and those not taking iron supplements. This study has increased our understanding of the patient subgroups at risk for anemia.

## Background

Chronic kidney disease (CKD) develops as a result of progressive loss of renal function due to structural or functional abnormalities of the kidney, and has important implications for health (KDIGO Work Group 2013). In 2013, CKD was identified as the 8th leading cause of death in Singapore (Ministry of Health, Singapore 2013). The incidence of CKD has been on the rise locally in Singapore as well as globally, causing it to become an increasing health concern (National Registry of Diseases Office, Singapore 2013; Ruggenenti et al. 2001). In 2010, there were 267 new cases of end-stage renal disease (ESRD) per million resident population (crude rate) in Singapore, up from 194 per million resident population in 1999 (National Registry of Diseases Office, Singapore 2013). The mortality and economic burden of the disease can be substantial (Valderrabano et al. 2001).

Anemia is a common complication that contributes to the burden of CKD (Basile 2007). It is diagnosed in adults and children >15 years old with CKD when the hemoglobin (Hb) concentration is <13.0 g/dL in male patients and <12.0 g/dL in female patients (KDIGO Anemia Work Group 2012). While the primary cause of anemia is the inadequate production of erythropoietin by the kidneys to support erythropoiesis (Ramanath et al. 2012), other factors may contribute to CKD-associated anemia, including decreased red blood cell life span, iron, folate and vitamin B12 deficiencies, blood loss, and accumulation of toxic inhibitors of erythropoiesis (McFarlane et al. 2008; Hsu et al. 2002).

Although anemia is common in CKD patients, it is variable in its time of presentation and severity among different individuals. Untreated anemia can also accelerate the decline in renal function by causing renal hemodynamic alterations and tissue hypoxia (Portolés et al. 2013; Iseki and Kohagura 2007). Patients with CKD and anemia often experience symptoms such as weakness, shortness of breath, and are at increased risk of cardiovascular diseases (CVD) and complications such as left ventricular hypertrophy (National Kidney Foundation 2006). This leads to higher consumption of health-care resources and increased risk of mortality (London et al. 2002).

Although full correction of anemia from low Hb concentrations have been shown to increase morbidity and mortality in CKD patients (Singh et al. 2006; Drüeke et al. 2006; Pfeffer et al. 2009), partial correction has been shown to improve cardiac function (Hayashi et al. 2000), cognitive function (Pickett et al. 1999) and quality of life in CKD patients (Revicki et al. 1995; Drüeke et al. 2006). It is thus necessary to identify predictors for anemia in CKD patients so that prevention and management strategies can be adopted early before complications develop. The third National Health and Nutrition Examination Survey (NHANES) study conducted in 1988–1994 showed a significant increase in the prevalence of anemia as patients’ glomerular filtration rate (GFR) fell below 60 mL/min (Hsu et al. 2002; Astor et al. 2002). Other studies identified sex, race, serum ferritin levels and comorbidities as predictors for anemia (Basile 2007; McClellan et al. 2004; Gilbertson et al. 2009; Al-Khoury et al. 2006).

However, most of these studies were conducted using the anemic CKD population from the US. The findings of these studies might not be representative of our local patient population due to differences in geographical location, lifestyle, racial and genetic make-up. Additionally, studies have shown that Hb concentrations vary between races, with African-American individuals consistently showing Hb concentrations 0.5 to 0.9 g/dl lower than Whites or Asians (Denny et al. 2006; Cresanta et al. 1987; Pan and Habicht 1991).

### Study aim

Risk factors of anemia in multi-ethnic CKD patients in Singapore are not yet well established and no studies have been done to assess potential local factors. As such, a case–control study involving non-dialysis CKD patients with and without anemia was conducted to determine clinical and/or demographics parameters that can predict the development of anemia.

## Results

### Clinical and demographic parameters

**Fig. 1:**
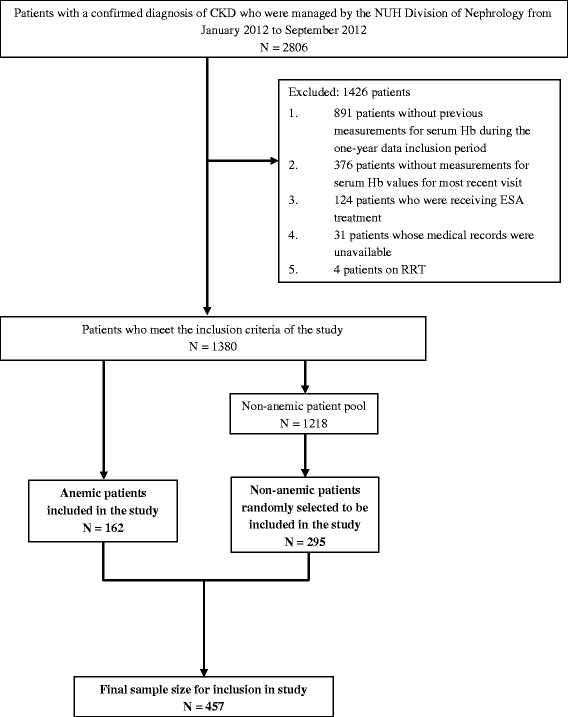
Patient recruitment chart

The patient recruitment process is illustrated in Fig. 1. A total of 2806 patients had clinic visits during the study period of January 2012 to September 2012. After screening, 1426 patients were excluded, and 1380 patients were found to be eligible for the study. Of these, 162 patients had Hb <10 g/dL and were thus classified as being anemic. All 162 patients were included in the study (cases). A random selection of 295 patients from the non-anemic pool was included into the study (controls). The final sample size for the study is 457 patients.

The patients’ demographic, clinical and laboratory data are presented in Table 1, and their medical and medication histories are presented in Table 2. The mean age of the patients was 67.0 ± 13.6 years and 53 % were male. It was found that 12.3 % of the patients had stage 1–2 CKD while 87.7 % of the patients had moderate to severe CKD (stage 3 and above). Majority of the patients had dyslipidemia (92.8 %), hypertension (89.3 %) and diabetes mellitus (64.6 %). The use of angiotensin receptor blockers (42.5 %), iron supplements (32.2 %) and angiotensin converting enzyme inhibitors (31.7 %) was common.

**Table 1:** Patients’ demographic, clinical and laboratory parameters

Parameters	Anemic patients		Non-anemic patients	
	N	Number (%) or mean ± SD	N	Number (%) or mean ± SD
Demographics				
Age, years	162	68.8 ± 13.2	295	66 ± 13.8
Gender				
(Female/Male)	162	91 (56.2) / 71 (43.8)	295	124 (42.0) / 171 (58.0)
Race				
Chinese/Malay/Indian/Others	162	99 (61.1) / 34 (21.0) / 16 (9.9) / 13 (8.0)	295	202 (68.5) / 56 (19.0) / 18 (6.1) / 19 (6.4)
Clinical data				
Weight, kg	67	61.8 ± 13.1	137	68.1 ± 15
Height, m	65	1.6 ± 0.1	135	1.6 ± 0.1
Body mass index, kg/m2	65	25.4 ± 4.8	133	27 ± 5.3
CKD stage				
Stage 1/2/3/4/5	162	3 (1.9) / 8 (4.9) / 29 (17.9) / 52 (32.1) / 70 (43.2)	295	45 (15.3) / 0 (0.0) / 127 (43.1) / 104 (35.3) / 19 (6.4)
Laboratory data (serum levels), units				
Current Hb, g/dL	162	9.2 ± 0.7	295	12.6 ± 1.7
Previous Hb, g/dL	162	9.9 ± 1.3	295	12.6 ± 1.7
Sodium, mmol/L	161	137.7 ± 3.8	292	138.1 ± 3.1
Potassium, mmol/L	161	4.5 ± 2.6	292	4.3 ± 0.5
Chloride, mmol/L	136	107 ± 5.8	257	106.1 ± 4
Bicarbonate, mmol/L	150	22.9 ± 3.9	270	24.7 ± 3.1
Urea, mmol/L	161	15.1 ± 8	292	10.5 ± 5.5
Creatinine, μmol/L	161	257 ± 188.7	293	174.3 ± 87.9
Calcium, mmol/L	150	2.2 ± 0.2	269	2.2 ± 0.1
Corrected calcium, mmol/L	99	2.3 ± 0.1	206	2.3 ± 0.1
Phosphate, mmol/L	148	1.3 ± 0.3	262	1.2 ± 0.2
Alkaline phosphatase, U/L	149	86.1 ± 58.5	262	73.4 ± 42.6
Albumin, g/L	145	34 ± 5.5	239	37.7 ± 4.2
Total cholesterol, mmol/L	128	4.6 ± 1.5	258	4.7 ± 1.3
Triglycerides, mmol/L	128	1.6 ± 0.8	258	1.8 ± 1.7
High-density lipoprotein, mmol/L	128	1.2 ± 0.4	257	1.2 ± 0.4
Low-density lipoprotein, mmol/L	128	2.7 ± 1.3	254	2.7 ± 1.1
White blood cells, x109/L	162	8.1 ± 4	295	7.8 ± 2.9
HbA1c, %	127	7 ± 1.5	193	7.4 ± 1.6
Iron, μmol/L	94	10.3 ± 5.7	80	11.4 ± 5.6
Ferritin, μg/L	97	397.9 ± 490.8	85	275.5 ± 520.0
Transferrin, mg/dL	94	186 ± 50.5	80	205.1 ± 53.6
Total iron binding capacity, μmol/L	94	48.4 ± 13.1	79	54.1 ± 12.7
Transferrin saturation, %	93	22.6 ± 11.9	79	22.2 ± 12.0
Intact parathyroid hormone, pmol/L	81	16.3 ± 15.4	129	11.9 ± 13.7
25-hydroxyvitamin D, μg/L	34	17.1 ± 11	60	22.4 ± 11.8
Folate, nmol/L	39	52.7 ± 71.2	44	27.8 ± 33.4
Vitamin B12, pmol/L	42	488.7 ± 260.4	50	450.6 ± 292.9

**Table 2:** Patients’ medical and medication histories (*N* = 162 for anemic patients, *N* = 295 for non-anemic patients)

Parameters	Number (%)	
	Anemic patients	Non-anemic patients
Medical conditions		
Dyslipidemia	150 (92.6)	274 (92.9)
Hypertension	149 (92)	259 (87.8)
Hematological disorders	146 (90.1)	55 (18.6)
Diabetes mellitus	112 (69.1)	183 (62)
Other cardiovascular disease	75 (46.3)	99 (33.6)
Ischemic heart disease	67 (41.4)	116 (39.3)
Cerebrovascular accident	47 (29)	51 (17.3)
Respiratory disorders	43 (26.5)	19 (6.4)
Heart failure	35 (21.6)	54 (18.3)
Gastrointestinal disorders	19 (11.7)	36 (12.2)
Endocrine disorders	5 (3.1)	15 (5.1)
Oncologic disorders	3 (1.9)	13 (4.4)
Immunological disorders	3 (1.9)	12 (4.1)
Medications		
Iron supplements	69 (42.6)	78 (26.4)
Angiotensin receptor blockers	59 (36.4)	135 (45.8)
Angiotensin-converting-enzyme inhibitors	38 (23.5)	107 (36.3)
Renalmin/Revalvite	33 (20.4)	44 (14.9)
Vitamin B12 supplements	30 (18.5)	31 (10.5)
Immunosuppressants	8 (4.9)	20 (6.8)
Fibric acid derivatives	5 (3.1)	22 (7.5)
Folate supplements	13 (8)	10 (3.4)

### Predictors of anemia status

A total of 21 potential predictors were identified after performing univariate logistic regression analyses. Backward elimination reduced this to 5 parameters. The potential predictors identified were previous Hb, CKD stage, hematological disorders, respiratory disorders and use of iron supplements. Gender but not race was found to be significant in univariate logistic regression analyses. However, this significance was lost after multivariate logistic regression analyses.

Since gender and race were found to be significant predictors of anemia in other studies (McClellan et al. 2004; Al-Khoury et al. 2006), the results of multivariate logistic regression analysis using the 5 predictors found in this study, together with gender and race, are presented in Table 3. The multivariate logistic regression analysis using the 5 predictors alone is similar and is given in the Supplementary information together with the results from the univariate logistic regression analyses (available upon request).

**Table 3:** Adjusted odds ratios of developing anemia (serum Hb <10 g/dL)

Parameters	Adjusted OR	*P*-value
	(95 % confidence interval)	
Demographics		
Gender		
Male	1.00 (Reference)	
Female	0.90 (0.44 – 1.87)	0.786
Race		
Chinese	1.00 (Reference)	
Malay	1.87 (0.77 – 4.56)	0.170
Indian	1.49 (0.42 – 5.34)	0.537
Others	2.16 (0.62 – 7.54)	0.228
Clinical data		
CKD stage		
Stage 1 & 2	1.00 (Reference)	
Stage 3	0.99 (0.23 – 4.19)	0.991
Stage 4	1.68 (0.41 – 6.86)	0.473
Stage 5	16.76 (3.49 – 80.52)	<0.001*
Laboratory data		
Previous Hb, g/dL	0.32 (0.23 – 0.44)	<0.001*
Medical conditions		
Hematological disorders	18.61 (8.44 – 41.07)	<0.001*
Respiratory disorders	4.54 (1.61 – 12.83)	0.004*
Medications		
Use of iron supplements		
No	1.00 (Reference)	
Yes	0.44 (0.21 – 0.93)	0.031*

* *P* < 0.05

As expected, previous Hb was predictive of anemia development (OR 0.32, 95 % CI 0.23-0.44, *p* < 0.001). In addition, the multivariate adjusted odds of anemia development were 16.8 times higher for a patient with stage 5 CKD (OR 16.76, 95 % CI 3.49-80.52, *p* < 0.001). Patients with haematological disorders (OR 18.61, 95 % CI 8.44-41.047, *p* < 0.001) and respiratory disorders (OR 4.54, 95 % CI 1.61-12.83, *p* = 0.004) also had higher odds of anemia. Patients had lower odds of anemia development if they were receiving iron supplements (OR 0.44, 95 % CI 0.21-0.93, *p* = 0.031).

## Discussion

This single-centre, retrospective case–control study evaluated the possible predictors of anemia development in patients with CKD. Anemia (defined as Hb <10 g/dL) was present in approximately one-third (35.4 %) of the 457 patients evaluated in the study. Although CKD-associated anemia can be easily diagnosed and is typically amenable to treatment (KDIGO Anemia Work Group 2012), it is known to be an adverse indicator for the progression of CKD and a critical risk multiplier for cardiovascular diseases. These complications associated with anemia can lead to an increase in mortality (Silverberg et al. 2009) and the large consumption of healthcare resources (London et al. 2002). Studies have also shown that CKD patients with Hb near normal levels experience functional improvements and significantly better quality of life (Moreno et al. 1996; Moreno et al. 2000). Hence, emphasis should be placed on the prevention of anemia development rather than treatment, since it plays a crucial role in slowing the progression of CKD and other related comorbidities (Rossert et al. 2005).

Previous studies have identified factors such as GFR, gender, race, iron administration and comorbidities *e.g.* diabetes mellitus as potential predictors for anemia in CKD patients (Hsu et al. 2002; Astor et al. 2002; McClellan et al. 2004; Gilbertson et al. 2009; Al-Khoury et al. 2006). Some results from this study were consistent with the previously-conducted studies where the potential for anemia development was found to be higher in patients with poorer renal function, lack of iron administration and presence of comorbidities. However, some differences in results were also observed.

### Previous hemoglobin concentration

**Fig. 2:**
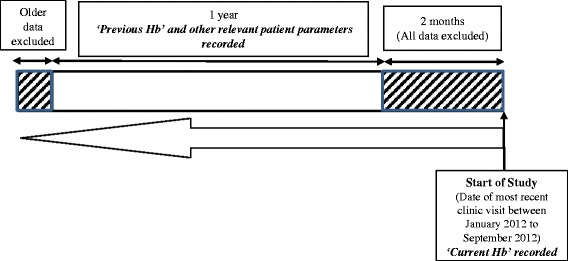
Method of data collection

The finding that previous Hb is important for predicting anemia is not unexpected and this supports the continual use of Hb as a parameter for evaluating future anemic status. However, while current KDIGO guidelines recommend that Hb be measured at least annually in non-anemic patients with stage 3 CKD and twice a year in those with stage 4–5 CKD (non-dialysis), the optimal frequency of monitoring is not yet known (KDIGO Anemia Work Group 2012). In this study, previous Hb was collected at least two months prior to the most recent visit and up to a year prior to this two-month period *i.e.* Hb values were obtained between two to 14 months from the most recent clinic visit (Fig. 2). The identification of previous Hb as an independent predictor of anemia development in this study suggests that a monitoring frequency of between two to fourteen months for Hb is appropriate for predicting anemia in this Asian CKD patient population, regardless of their clinical history. However, this time interval is rather wide and the effect of Hb variations within this time period is unknown. Hence, further research on the use of previous Hb values from different time periods to determine how dated Hb levels can be before they lose their predictive value, as well as the effect of Hb variations during this time period are needed. Knowledge of these may shed some light on the optimal frequency of Hb measurements and monitoring.

### CKD stage

The risk of anemia development was found to increase significantly as CKD worsens. This finding is consistent with previous reports from the NHANES III study which showed that the prevalence of anemia increases as eGFR falls (1 % at 60 ml/min/1.73 m^2^, 9 % at 30 ml/min/1.73 m^2^ and 33 % at 15 ml/min/1.73 m^2^) (McFarlane et al. 2008; Hsu et al. 2002; Coresh et al. 2003). In this present study, stage 5 CKD patients were 16.8 times more likely to develop anemia as compared to patients with stage 1 and 2 CKD. Deterioration of renal function is accompanied by a reduction in erythropoietin production by the kidneys, which accounts for approximately 90 % of erythropoietin production in the body (Moore and Bellomo 2011). The loss of erythropoietin results in decreased red blood cell production that causes anemia. This explains why worsening renal function increases the risk of anemia development.

### Comorbidities

While diabetes was found to be an independent predictor of Hb levels in a study involving 468 CKD patients (Al-Khoury et al. 2006), it was not found to increase the odds of anemia development in this study. Instead a history of hematological and respiratory disorders conferred greater odds of anemia in our patient population.

A total of 201 patients (44.0 %) had hematological disorders, which included alpha-thalassemia and previous history of anemia or episodes of anemia (within the one-year period of data collection) with etiologies other than CKD. Patients with a history of such disorders are expected to have greater odds for developing anemia subsequently, as they are already at risk. This suggests that any CKD patient who presents with a hematological disorder should be more closely monitored for anemia. Similarly, patients with respiratory disorders such as chronic obstructive pulmonary disease (COPD) and asthma had higher odds of developing anemia. A recent study found a high prevalence of CKD in patients with COPD, and chronic inflammation associated with COPD is considered to contribute to anemia development (Incalzi et al. 2010). Optimal management of comorbid respiratory conditions may therefore reduce the odds of developing anemia.

### Use of iron supplements

This study showed that patients without iron supplementation had approximately twice the odds of acquiring anemia compared to patients who do. This was similarly found in a study conducted in hemodialysis patients where intravenous iron administration was associated with higher Hb (Gilbertson et al. 2009). Iron deficiency is common in patients with CKD (Wittwer 2013). This is often due to poor dietary intake or bleeding, eventually leading to a reduction in formation of red blood cell Hb, causing hypochromic microcytic anemia (Fishbane and Singh 2009). Thus, iron supplementation is necessary for optimal management of anemia and can in fact attenuate the progression of anemia in CKD (Kim et al. 2011).

### Gender and race

Gender and race have been found to have significant associations with anemia in CKD patients in other studies (McClellan et al. 2004; Al-Khoury et al. 2006). McClellan *et al.* (McClellan et al. 2004) reported higher odds of developing anemia in female patients. Differences in the prevalence of anemia by racial groups such as in Caucasian, African-American, Native American, Hispanic and Asian patients were also evident. However, such gender and race differences were not identified in this study. Female patients in the study by McClellan *et al.* had lower Hb concentrations than male patients, which likely explain why females had greater risk of developing anemia (McClellan et al. 2004). Although the univariate analysis showed female patients having higher odds of developing anemia in this study, this statistical significance was lost after adjustment. As our multivariate analysis already accounted for the patients’ Hb concentrations by taking their previous Hb into consideration, this could explain the lack of association between gender and anemia in this study. The Singapore population is made up of 3 major racial groups, namely Chinese, Malay and Indians. This is vastly different from the ethnic composition of the US population. This study did not show patients from either racial group having higher odds of developing anemia. However, this should be further confirmed by future studies with larger sample size.

### Limitations

This study is not without limitations. Firstly, this was a single-center study and the number of anemic patients (162) in our study was small. As such we could not perform external validation of the model that we had constructed. This could have potentially introduced biases into the final analysis and limited the generalizability of the findings to the entire anemic CKD population in Singapore.

Next, the cross-sectional nature of the study limits the ability to show any direct cause and effect relationship between the parameters collection and anemia development. As only two sets of Hb level readings were collected, it was not possible to determine any trend or monitor for fluctuations in Hb level readings across the one-year data collection period of each patient. Missing data from the electronic medical records also posed challenges in collecting complete information for each patient. Additionally, the inclusion of patients with hematological disorders and previous history of anemia, albeit not secondary to CKD, could have confounded the results. Other novel markers of anemia such as hepcidin were also not obtained as they were not routinely measured in our clinical setting.

Despite the limitations, this study has several advantages over existing data, such as the inclusion of a sizeable local population of non-dialysis CKD patients naïve to ESAs. These findings may present an opportunity for earlier detection and intervention in patients who present with the predictors identified in this study before anemia develops. Future, larger studies will need to be conducted to determine the optimal Hb level at which correction of these risk factors should be initiated. This would allow for a more effective management of anemia and potentially improved outcomes in the local CKD population. Additionally, prediction algorithms can be developed in the future to better predict the risk of anemia development when patients present with these predictors, and improve the study’s applicability in the clinical setting.

## Conclusion

This study found that the probability of anemia development was greater in our local CKD patients with lower previous hemoglobin, more advanced CKD, presence of haematological and respiratory disorders, as well as not using any iron supplements.

## Methods

### Study design

This single-centre, retrospective, case–control study was conducted at the National University Hospital (NUH), Singapore. Approval from the National Healthcare Group Domain-Specific Review Board, our local Institutional Review Board was obtained. A list of patients with a confirmed diagnosis of CKD and were managed by the NUH Division of Nephrology from January 2012 to September 2012 was obtained for this study.

All adult (≥21 years old) non-dialysis patients with stage 1–5 CKD, who had at least two serum Hb readings during the data collection period (described in Fig. 2) were included in the study, regardless of their Hb levels. Patients were excluded if they were receiving renal replacement therapy (RRT), their serum Hb was not measured at their most recent clinic visit, their medical records were unavailable or if they were receiving erythropoiesis-stimulating agents. Informed consent was not necessary as this was a retrospective, case–control study.

### Data collection

The method of data collection is illustrated in Fig. 2. Data were collected and reviewed from the patients’ electronic medical records. Patients were screened to determine if both their ‘Current Hb’ and ‘Previous Hb’ levels were available. ‘Current Hb’ refers to the serum Hb at the most recent clinic visit and was used to determine whether a patient has anemia. ‘Previous Hb’ refers to the serum Hb measured at the preceding clinic visit and was expected to be an important predictor of anemia. All other parameters from the patients’ preceding clinic visit that were considered to be possible predictors of anemia were also collected. These parameters included demographic characteristics such as age, sex and race; clinical data such as body mass index (BMI), CKD stage; medical and medication histories; and laboratory data such as serum electrolytes, urinary and hematologic parameters. The parameters were evaluated to determine if they had any value in predicting the development of anemia in the patients.

All data within two months prior to the most recent clinic visit were excluded and only data that were up to one year prior to the two-month period were included. The two-month period for data exclusion was selected as the body’s circulating red blood cells (RBCs) would need to be depleted before any changes in Hb can be detected. This two-month period would also be representative of the lifespan of RBCs in CKD patients (Ly et al. 2004; Nurko 2006), which is shorter than the 120 days RBC life-span of a normal, healthy individual. The data inclusion period was also limited to no longer than a year as older data would have less predictive value on anemia development. Hence, if Hb was measured multiple times in the span of the one-year period, only the most recent results were included. By comparing changes between the previous and current Hb levels, the prediction models constructed can estimate the patient’s likelihood of developing anemia at least two months later.

### Data processing

Using the collected ‘Current Hb’ levels, each patient was classified as either being anemic or non-anemic using a threshold level of 10 g/dL. A patient was classified as being anemic and assigned as a “case” group if his/her current Hb level was <10 g/dL. This is based on the KDIGO guidelines (KDIGO Anemia Work Group 2012) and the US FDA’s recommendation to initiate ESAs in CKD patients when the Hb level is <10 g/dL (FDA Drug Safety Communication 2007). Patients whose Hb was ≥10 g/dL) were considered non-anemic and were assigned as “controls”. The CKD stage for each patient was determined based on their eGFR calculated using the four-variable MDRD equation for standardized creatinine.

Multiple imputation was used to fill in all missing data, as this would yield less biased results compared to using complete case analysis (White and Carlin 2010). This was done *via* sequential imputation using chained equations. As the data to be imputed were continuous and may have non-normal distributions, the predictive mean matching (PMM) method was selected (Vink et al. 2014). All other covariates, including the outcome parameter (*i.e.* current Hb), were included in the imputation model to avoid bias. A random seed of 123 was used, and 100 imputations were performed.

### Statistical analysis

The correlation between the development of anemia and different patient parameters was determined by performing univariate logistic regression analyses. Odds ratios (OR) were calculated to estimate the percentage change in risk of anemia development. Parameters with p-values <0.05 were considered statistically significant. These parameters were subsequently used for multivariate logistic regression analysis, and backward elimination was used to remove parameters with p-values ≥0.05 in the multivariate logistic regression model. In backward elimination, parameters with the largest p-values were removed at each stage until all the remaining parameters had p-values <0.05. Adjusted ORs were computed for each of the remaining parameters. All statistical analyses were performed using Stata/SE Version 13.1 (College Station, TX, USA).

## Abbreviations

BMI: body mass index
CI: confidence interval
CKD: chronic kidney disease
COPD: chronic obstructive pulmonary disease
ESA: erythropoiesis-stimulating agent
Hb: hemoglobin
MDRD: Modification of Diet in Renal Disesae
NHANES: National Health and Nutrition Examination Survey
NUH: National University Hospital
OR: odds ratio
PMM: predictive mean matching
RBC: red blood cell
RRT: renal replacement therapy
US: United States
